# Discordant anti-müllerian hormone (AMH) and follicle stimulating hormone (FSH) among women undergoing in vitro fertilization (IVF): which one is the better predictor for live birth?

**DOI:** 10.1186/s13048-018-0430-z

**Published:** 2018-07-16

**Authors:** Shunping Wang, Yi Zhang, Virginia Mensah, Warren J. Huber, Yen-Tsung Huang, Ruben Alvero

**Affiliations:** 1 Brown University Warren Alpert Medical School, Providence, RI 02912 USA; 2 Women and Infants Fertility Center, Women and Infants Hospital of Rhode Island, 101 Dudley Street, Providence, RI 02905 USA; 3 Brown University School of Public Health, Providence, RI 02912 USA; 4 Institute of Statistical Science Academia Sinica, 128 Academia Road Sec. 2, Taipei, 11529 Taiwan

## Abstract

**Background:**

This study sought to clarify the roles of Anti-müllerian hormone (AMH) and follicle stimulating hormone (FSH) in predicting live birth, especially in patients with discordant AMH and FSH. A large IVF data set provided by eIVF®, consisting of 13,964 cycles with AMH, FSH, age, BMI, and birth outcomes were evaluated. Patients were categorized into four groups: Good prognosis group (AMH ≥1 ng/ml; FSH < 10 mIU/ml), Poor prognosis group (AMH < 1 ng/ml; FSH ≥10 mIU/ml), Reassuring AMH group (AMH ≥1 ng/ml; FSH ≥10 mIU/ml), and Reassuring FSH group (AMH < 1 ng/ml; FSH < 10 mIU/ml). The interaction between AMH, FSH, and their impact on live birth rate among these four groups was evaluated using Generalized Additive Mixed Modeling (GAMM).

**Results:**

Analysis revealed a nonlinear relationship of AMH and FSH with live birth rate among all ages. Among the four groups, the good prognosis group had the highest live birth rate while the poor prognosis group had the lowest live birth rate (29.3% vs 13.1%, *p* < 0.005). In the discordant groups, the live birth rate of the reassuring AMH group was significantly higher than the reassuring FSH group (22.8% vs 15.6%, *p* < 0.005).

**Conclusions:**

Although both FSH and AMH are widely use to assess the ovarian reserve in women undergoing evaluation for infertility, AMH appears to be superior to FSH among all age groups. This is particularly important for patients with discordant AMH and FSH where reassuring AMH is a better clinical predictor of cycle success.

**Electronic supplementary material:**

The online version of this article (10.1186/s13048-018-0430-z) contains supplementary material, which is available to authorized users.

## Background

In women undergoing evaluation for infertility, ovarian reserve testing with anti-müllerian hormone (AMH) and follicle stimulating hormone (FSH) provides important prognostic information regarding reproductive outcomes. AMH is a peptide hormone produced by granulosa cells of early antral follicles and can be collected at any point during a woman’s menstrual cycle [1–3]. Although no established cutoff for normal and abnormal AMH exists, it is generally accepted that AMH > 0.8–1.0 ng/ml are suggestive of normal ovarian reserve [4]. FSH is a hormone produced by the anterior pituitary and when elevated above 10 mIU/ml, is suggestive of diminished ovarian reserve [5]. Both markers are affected by a woman’s age: AMH decreases as age increases, while FSH increases as age increases. The American Society for Reproductive Medicine considers evaluation of both serum methods acceptable measures of ovarian reserve [6]. Although AMH and FSH are generally accepted as useful in predicting response to ovarian stimulation, existing evidence is controversial regarding the utility of both markers for the prediction of live birth [4, 5, 7–14].

In a retrospective review of 76 in vitro fertilization (IVF) cycles, Barad et al. found AMH to be a superior predictor of clinical pregnancy outcome compared to FSH [15]. Similarly, Nelson et al. evaluated 340 patients undergoing first IVF or Intracytoplasmic Sperm Injection (ICSI) cycles and found that AMH predicts live birth and anticipated oocyte yield better than FSH and age [16]. Another retrospective analysis comparing multiple markers of ovarian reserve determined that AMH, antral follicle count, and quantity of oocytes retrieved were the most reliable predictors of live birth [17]. These studies, though compelling, are limited by small sample sizes and stringent inclusion criteria which limits their external validity. The question, therefore, of which ovarian reserve marker is a better predictor of live birth remains unanswered, leaving infertility specialists with limited evidence to guide their treatment decisions.

Clinicians additionally often encounter a discrepancy between the two markers—a situation which can affect the interpretation of a woman’s likelihood of live birth. Leader et al. showed a frequency of AMH and FSH discordance of as many as 1 in 5 evaluations for female infertility [18]. In a small retrospective study, having an elevated FSH (> 10 mIU/ml) but reassuring AMH (> 0.6 ng/ml) was found to be significantly associated with higher oocyte yield, greater number of day 3 embryos, and lower cycle cancellation rates compared to women with random AMH levels < 0.6 ng/ml. Clinical pregnancy rate among this group was likewise higher, but the difference was not statistically significant [19]. Gleicher et al. similarly reported that among 115 female infertility patients with discordant AMH and FSH (normal age specific AMH with abnormal FSH), oocyte yield was diminished compared to their AMH/FSH concordant counterparts (normal age specific AMH and FSH) [20]. Still, when discordant results are encountered, there is a paucity of data regarding the prognostic relationship between AMH and FSH.

We sought to investigate this question of the clinical utility of AMH and FSH in a retrospective analysis of the eIVF® database, a multi-center dataset that encompasses over 140,000 cycles of assisted reproduction at over 60 fertility centers. The main objective of this study is to evaluate whether AMH or FSH is a better predictor of live birth among infertility patients of differing ages. Additionally, when AMH and FSH markers are discordant and confer potentially conflicting prognostic values, we determine which marker is a more reliable estimate of successful pregnancy outcome.

## Methods

### Patient selection

eIVF® is an electronic medical record software for clinical IVF settings designed by PracticeHwy.com (Dallas, Texas). The software package includes portals integrating clinical, administrative, and financial information. The dataset we obtained consisted of 144,044 fresh cycles from 60 centers in the United States from 2000 to 2016. Evaluation of this comprehensive de-identified dataset was determined to be exempt by the Women and Infants Institutional Review Board.

**Fig. 1:**
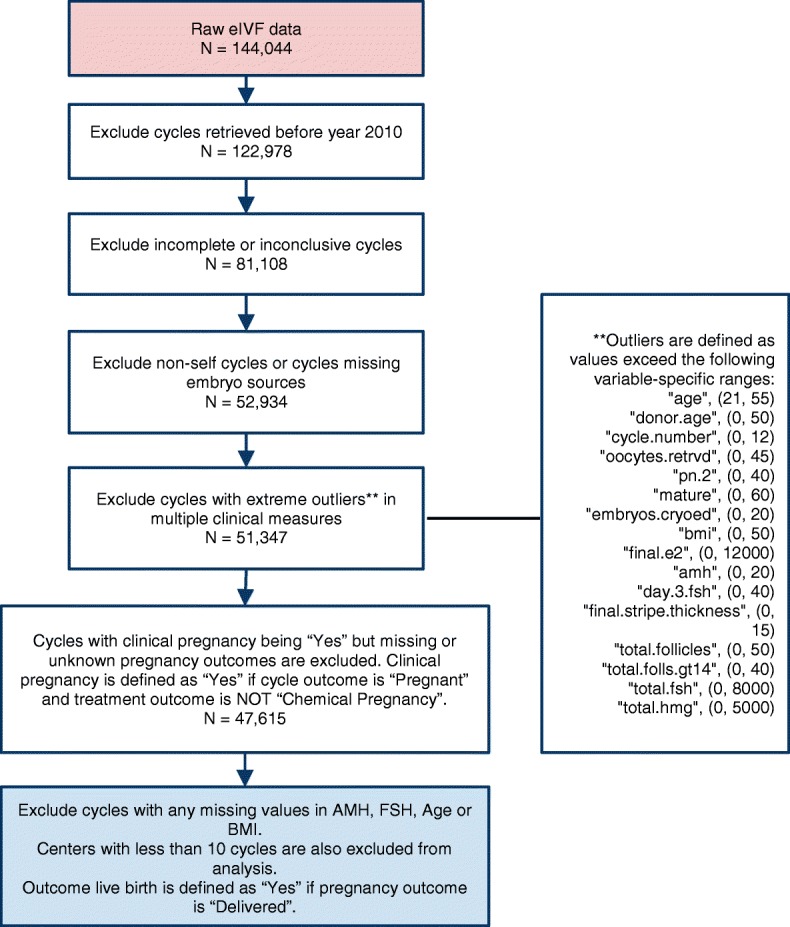
CONSORT diagram for data preparation process for analysis

Figure 1 shows our CONSORT diagram for data processing. We excluded cycles which were incomplete, were non-autologous donor cycles, had unknown or missing cycle information, or contained outlier variables. Centers with less than 10 cycles were also excluded. Following application of these exclusion criteria, only 47,615 cycles remained in the dataset. Of note, since AMH has only been adopted in clinical use in the past few years, most cycles before 2010 were excluded because of missing AMH values. Thus our final dataset contained 13,790 autologous IVF cycles with known AMH, FSH, and confirmed determination of live birth.

The 13,790 cycles included for analysis were further subdivided into four groups using AMH = 1.0 ng/ml and FSH = 10.0 mIU/ml as cutoff values for normal/reassuring testing. Groups I and II represent a patient population with concordance between their AMH and FSH results. Group I included cycles from all good prognosis patients with AMH greater than or equal to 1.0 ng/ml and FSH less than 10 mIU/ml. Group II included cycles from patients considered poor responders based on AMH less than 1.0 ng/ml and FSH greater than or equal to 10 mIU/ml. Groups III and IV represent a patient population with discordance between their ovarian reserve markers. Group III included the cycles with AMH less than 1.0 ng/ml and with FSH less than 10 mIU/ml, while Group IV included cycles with AMH greater than or equal to 1.0 ng/ml and with FSH greater than or equal to 10 mIU/ml (Table 1). Our primary outcome of interest was live birth per cycle initiated.

**Table 1:** Demographic characteristics and live birth rates of all four groups

All		Concordant		Discordant		
		Group I: Good Prognosis (AMH ≥ 1 & FSH < 10)	Group II: Poor Prognosis (AMH < 1 & FSH ≥ 10)	Group III: Reassuring FSH (AMH < 1 & FSH < 10)	Group IV: Reassuring AMH (AMH ≥ 1 & FSH ≥ 10)	
N	13,790	7997	1717	3271	805	
	Mean (SD)	Mean (SD)	Mean (SD)	Mean (SD)	Mean (SD)	*P*-values
Age	35.4 (4.7)	34.1 (4.5)	38.0 (4.1)	37.3 (4.3)	35.6 (4.4)	< 0.001
BMI	25.9 (6.0)	26.0 (6.0)	25.1 (5.2)	26.6 (6.3)	24.4 (4.9)	< 0.001
# of embryos transferred	1.7 (1.1)	1.8 (1.0)	1.5 (1.3)	1.8(1.2)	1.9 (1.2)	< 0.001
E2	2261 (1485)	2676 (1556)	1370 (973)	1690 (1151)	2240 (1243)	< 0.001
FSH (mIU/ml)	7.6 (3.8)	6.2 (2.0)	14.0 (4.4)	6.6 (2.2)	12.2 (3.1)	< 0.001
AMH (ng/ml)	2.4 (2.7)	3.6 (2.9)	0.4 (0.3)	0.5 (0.3)	2.3 (1.7)	< 0.001
Live Birth (%)	23.5%	29.1%^a,b,c^	12.8%^d, e^	15.4%^f^	22.7%	< 0.001

^a^Group I vs Group II. *p*-value < 0.001

^b^Group I vs Group III. *p*-value < 0.001

^c^Group I vs Group IV. *p*-value < 0.001

^d^Group II vs Group III. *p*-value 0.013

^e^Group II vs Group IV. *p*-value < 0.001

^f^Group III vs Group IV. *p*-value < 0.001

### Statistical analysis

Generalized additive mixed models (GAMM) were used to investigate the nonlinear fixed effects of AMH and FSH on live birth rate using penalized spline [21], while adjusting for the random effects of centers. AMH and FSH levels were transformed into log-scale before fitting the models because of their highly skewed distributions in our sample, and a small value, 0.7 was added to AMH and FSH levels before transformation to avoid taking logarithm of zero. GAMM were fit to delineate the marginal effects of AMH and FSH on live birth rate, adjusting for age. The joint effects of AMH and FSH were further characterized using two-dimensional spline under GAMM. The two-dimensional splines with AMH-by-FSH interaction and without were both explored to investigate the joint effects of AMH and FSH. All models were fitted through maximizing a penalized log-likelihood using R package mgcv. Based on the fitted models, we were able to predict the probability of live birth for a certain patient given one’s AMH, FSH and age. To visualize the dose-response relationship of AMH and/or FSH with respect to the probability of live birth, we plotted predicted probabilities given the corresponding AMH and FSH under each model.

## Results

Table 1 presents the baseline characteristics of the four groups based on our previously defined cutoffs. The live birth rate for good prognosis patients (Group I) was significantly higher than patients with poor prognosis (Group II) (29.1% vs 12.8%; *p* < 0.05). Among the two discordant groups, patients with reassuring AMH (Group IV) had significantly higher live birth rate compared to patients with reassuring FSH (Group III) (22.7% vs 15.4%, *p* < 0.05).

**Fig. 2:**
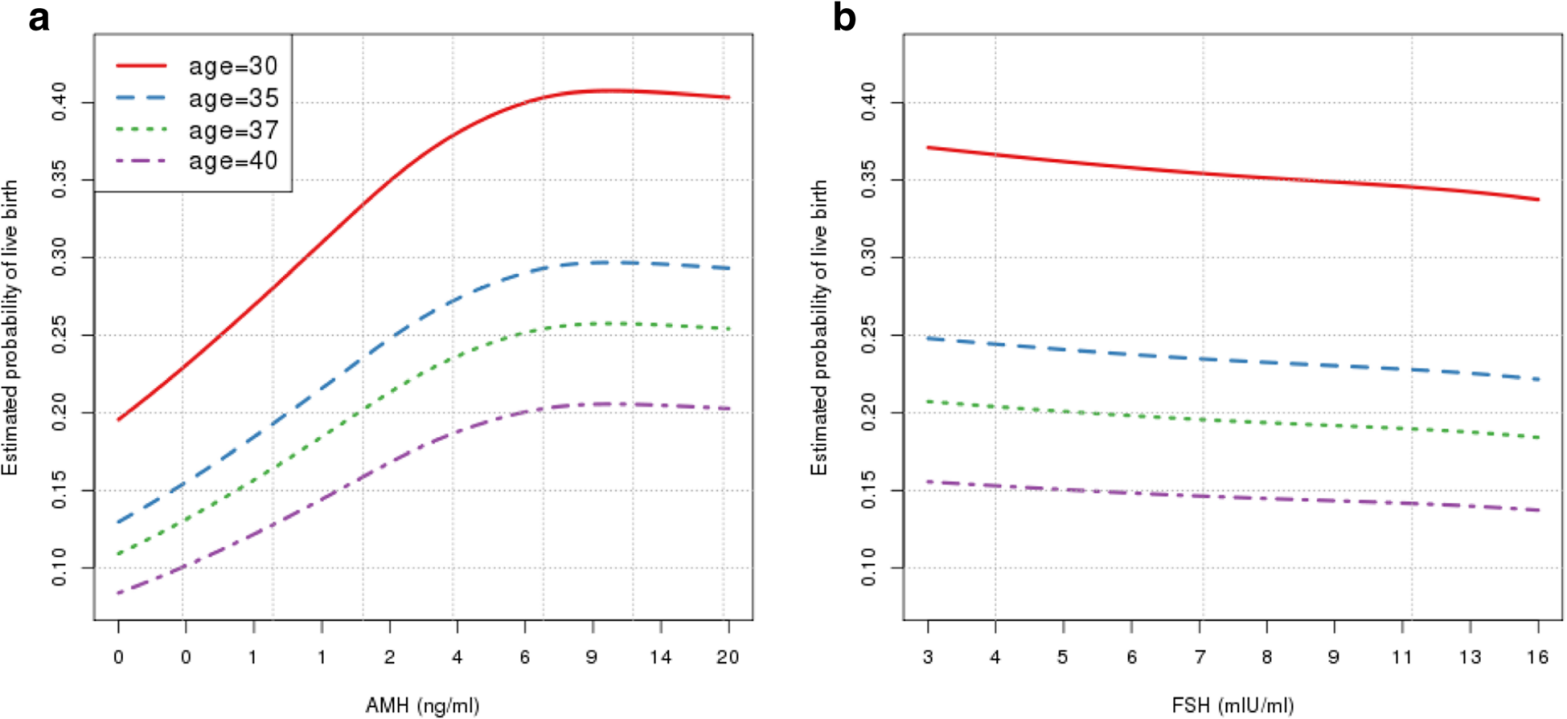
Estimated marginal dose-responsive relationships between estimated live birth rates and **a** AMH and, **b** FSH for patients of age 30, 35, 37, and 40 years old by generalized additive mixed models

Figure 2a and b show the GAMM established to predict the live birth rate using AMH and FSH respectively among patients of age 30, 35, 37, and 40 years old. Among all ages examined for AMH, there was a positive dose-response relationship between AMH and probability of live birth (Fig. 2a). Similarly, among all ages examined for FSH, there was a negative dose-response relationship between FSH and live birth (Fig. 2b), although not as significant as AMH. As AMH approached 6 ng/ml across all ages, there was a plateau in the estimated likelihood of live birth.

**Fig. 3:**
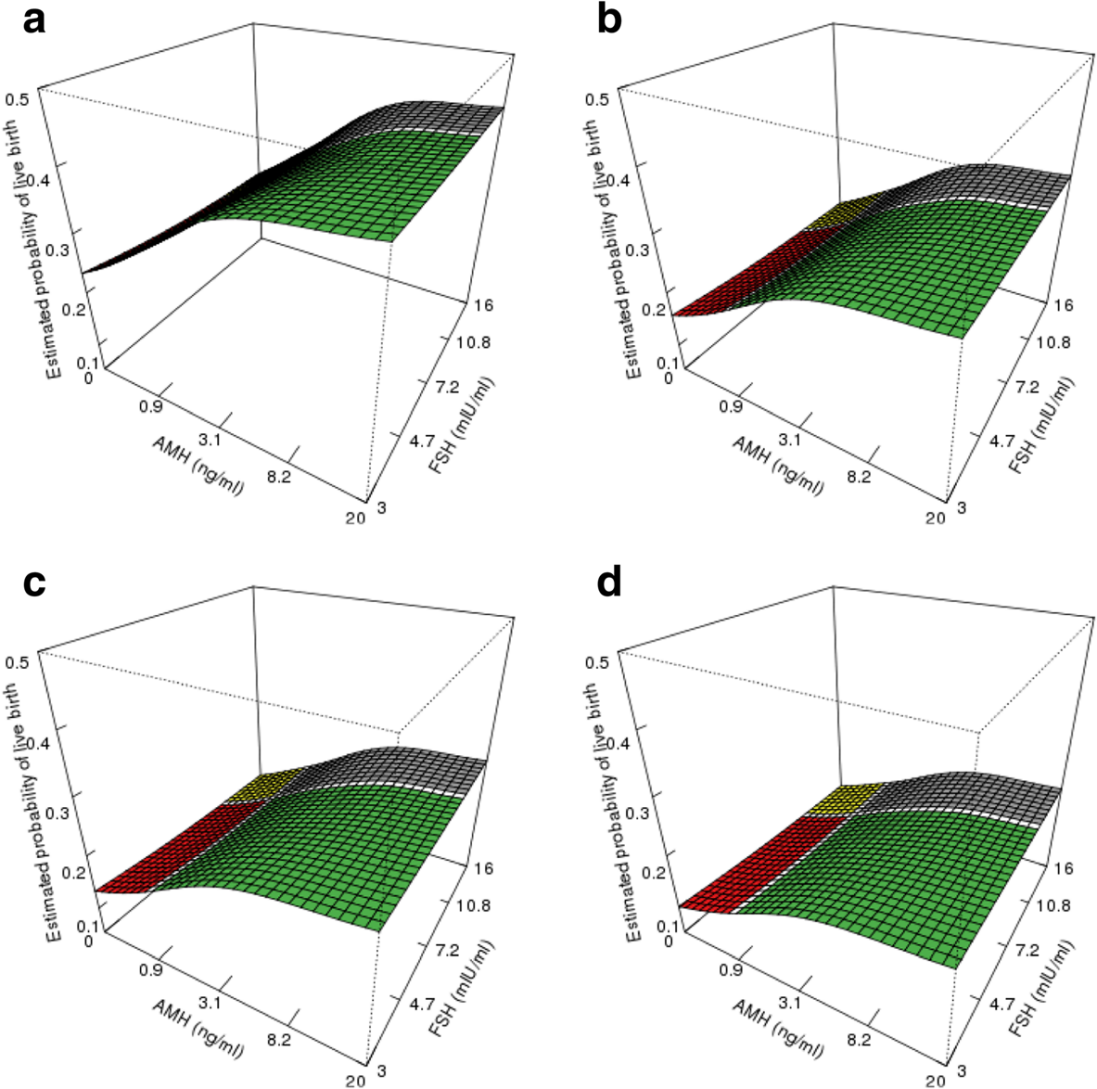
Estimated joint effects of AMH and FSH on live birth rates by two-dimensional generalized additive mixed models for patients from different age groups. **a** 30 years old, **b** 35 years old, **c** 37 years old, and **d** 40 years old. Green: good prognosis (AMH ≥ 1 ng/ml & FSH < 10 mIU/ml); Yellow: poor prognosis (AMH < 1 ng/ml & FSH **≥** 10 mIU/ml); Red: FSH reassuring group (AMH < 1 ng/ml & FSH < 10 mIU/ml); Grey: AMH reassuring group (AMH ≥ 1 ng/ml & FSH > 10 mIU/ml)

Figure 3 demonstrates our model for the joint effect of AMH and FSH on live birth rate. The two horizontal axes represent AMH and FSH values evenly spaced on log-scale, and the vertical axis indicates the estimated live birth rates based on two-dimensional GAMM. The predicted birth rates for patients with age 30, 35, 37, and 40 years old are shown in Fig. 3a, b, c, and d respectively. Consistent with the prior trend, the estimated probability of live birth decreases as age increases, given the same AMH and FSH. Within each figure panel of specified age, the predicted live birth probability ascends rapidly with AMH when AMH is less than 8.2 ng/ml for fixed FSH. In comparison, for any given AMH value, the estimated live birth probability only decreases slightly as FSH increases from the lowest truncated value to the highest. In other words, the joint effect of AMH and FSH is dominated by that of AMH. The three-dimensional graphs provide a comprehensive visualization of dose-response relationship between any combination of AMH, FSH, and live birth rate. The joint effect analysis indicates that AMH is a more reliable predictor of live birth rate than FSH. Particularly in the discordant groups, a reassuring AMH (grey region) suggests a better likelihood of live birth compared to reassuring FSH (red region). Consistent with the trend observed in marginal models, higher AMH has a positive effect on live birth success rate while higher FSH and age demonstrate negative effects.

## Discussion

To our knowledge, this is the first comprehensive analysis of the clinical utility of AMH and FSH with a sample size close to 14,000 cycles, with live birth as the primary outcome. AMH and FSH are widely accepted as predictors of ovarian response to stimulation with exogenous gonadotropins and therefore provide valuable prognostic clinical information prior to an IVF cycle start. Previous studies have reported on the utility of AMH and/or FSH as predictors of IVF cycle success defined by oocyte yield [22–24], number and quality of embryos [24], clinical pregnancy rate [22, 24], and live birth [17, 24, 25]. Our analysis advances these previous findings by suggesting that while both markers confer some prognostic value to the prediction of live birth, AMH is superior to FSH among all age groups. This is suggested by two principal findings in Fig. 3. First, the fact that irrespective of FSH value, a low AMH confers a lower likelihood of live birth among young patients. This live birth likelihood is even lower for older patients with low AMH. Additionally, in patients with high FSH, a high AMH rescues live birth probability (i.e. > 20%) across all age groups. Both findings suggest AMH is the more important determinant of pregnancy outcome than FSH.

Unsurprisingly, our study suggests live birth rates are highest in good prognosis cycles (Group I) and lowest in poor prognosis cycles (Group II). Prediction of cycle success is more difficult when AMH and FSH are discordant. In the 13,964 cycles we analyzed, AMH and FSH levels were discordant (Group IIII: AMH **≥**1 ng/ml and FSH **≥**10 mIU/ml and Group IV: AMH < 1 ng/ml and FSH < 10 mIU/ml) in 30% of cycles, compared to a 20% discordance between AMH and FSH in over 5300 women reported by Leader et al. Although this study clearly describes a high rate of discordance between AMH and FSH, it is limited by the absence of clinical outcomes. Gleicher et al. reported on the impact of AMH and FSH discordance on oocyte yield in a small prospective study of 350 IVF cycles [20]. In the discordant population, a normal AMH with abnormal FSH predicted a higher oocyte yield than a normal FSH with abnormal AMH. Although there were age dependent discrepancies, AMH was found to be a better clinical predictor of oocyte yield. In our study, a reassuring AMH predicted a higher live birth rate among discordant cycles, likewise suggesting that a normal AMH is a better clinical predictor of cycle success when AMH and FSH are discordant. We also examined the proportion PCOS, male factors, and the protocol types among the four groups (Additional file 1: Appendix 4, Table S1), but the role of these factors in the AMH/FSH-live birth rate association (e.g., as mediators, confounders, or effect modifiers) may require additional research.

Conventionally logistic regression models consisted of the first-order main effects of clinical measures are used to investigate the clinical utility of AMH and FSH in predicting IVF success rate. These parametric approaches make several assumptions about the data, such as underlying linear relationship and normally distributed errors between the predictors and outcomes, which may not accurately reflect the nature of the clinical measures. In this study we utilized a semiparametric regression modeling, penalized spline regression, to reduce the assumed linear relationship between predictors and outcome. The piecewise continuous polynomials, or splines, when combined with mathematical penalization, should provide a superior overall fit of the data compared to a conventional parametric approach. In addition, since our study sample are pooled from 26 IVF centers across the U.S., we also adjust for the center-level heterogeneity by including random intercept effects for each center in all models. Models without adjusting centers are shown in Additional file 1: Appendix 1. Our results suggest a nonlinear relationship of AMH and live birth rate among all ages. Once AMH levels reach a certain threshold, the live birth rate plateaus and further increases in AMH do not significantly increase the likelihood of live birth. Similarly, FSH demonstrates a nonlinear relationship with live birth rate: once FSH levels increase to a certain threshold, live birth rates decline for patients of all ages. These nonlinear relationships between AMH, FSH, and live birth mirror what clinicians often encounter in practice, but more importantly suggest that the ovarian reserve markers are associated with live birth in an age dependent manner. The statistical approach used in our study to evaluate AMH and FSH is flexible in characterizing non-linear dose-response relationship between predictors and outcomes, and thus provides an alternative analysis tool that could have been neglected in existing literature.

The marginal dose-response relationship of AMH or FSH with live birth rate (Fig. 2a and b), however, should be interpreted with certain caveats. For example, the marginal effect of AMH did not account for the effect contributed by FSH, and the high correlation of AMH and FSH may exert an undue influence, i.e., confounding on the AMH-live birth association. To address this issue, we further characterized the joint effect of AMH and FSH using GAMM with two dimensional splines where we were able to investigate the effect of one marker by adjusting for the other one. We also explored the possibility of expanding the above models to include potential confounder BMI, which resulted in very similar results (Additional file 1: Appendix 2). We applied the above prediction models to an internal dataset from patients at our center to test the model validity. The Receiver Operating Curve area under curve calculation equaled 0.67, suggesting age, AMH, and FSH alone are perhaps not sufficient to accurately predict the IVF success rate. Our group is therefore currently working on a more sophisticated model which incorporates demographic information and treatment outcomes, in order to better predict the likelihood of success with IVF and facilitate individualized patient counseling.

Our study has several inherent limitations. We could not control for the type of AMH or FSH assays used given the diversity of geographic areas encompassed and centers queried. Thus, there may be some variation to the results, besides the adjusted center-level differences, that we are unable to account for due to the differences in assay sensitivity and specificity. Another limitation is that poor prognosis patients may contribute a large proportion of cycles in this dataset: in other words, since poor prognosis patients are more likely to undergo multiple cycles before achieving a live birth, they may be more heavily represented in this dataset. This will inevitably lead to an underestimation of the ability of both markers to predict live birth likelihood. As an attempt to address this limitation, we re-performed the analysis presented but on a subset of population (*n* = 9532) consisted of only the earliest cycle from each patient and the results are similar (Additional file 1: Appendix 3). In addition, the information on day of embryo transfer (Day 2, Day 3, or Day 5) is not available in this database. It is possible that good prognosis and AMH reassuring groups may have higher percentage of Day 5 embryo transfer, although this should not affect our results. Lastly, although the eIVF database contains live birth rates for IVF cycles from 2000 to 2016, we only included cycles from 2010 to 2016 in our statistical analysis as AMH became a widespread test for ovarian reserve in 2010. Conversely, there are multiple strengths to this study including its large, heterogeneous patient population, geographic diversity, and comprehensive timeframe of IVF treatment cycles assessed. Furthermore, this study is unique in that it may be the largest study of its kind to evaluate live birth in a population of women in whom both AMH and FSH results were either concordant or discordant.

## Conclusion

In conclusion, the ovarian reserve markers AMH and FSH are both associated with live birth probability, although AMH appears to be a stronger predictor especially in situations of discordant results. Although both demonstrate clear clinical utility for prognosis prediction in infertility patients, either marker evaluated alone or taken together are insufficient to predict a patient’s likelihood of live birth. Prediction models which incorporate these markers in addition to other patient demographics and treatment response information are needed to provide accurate prognostic guidance for infertility specialists to facilitate patient counseling.

## Additional file

Additional file 1: **Figure S1.** Estimated generalized additive mixed models (GAMMs) on different ages without adjusting for centers. A) AMH, and B) FSH. **Figure S2.** Joint model of AMH and FSH on predicting live birth rates without adjusting for centers. A) 30 year old, B) 35 year old, C) 37 year old, and D) 40 year old. **Figure S3.** Estimated generalized additive mixed models (GAMMs) on age and BMI. A) AMH, and B) FSH. **Figure S4.** Joint effect of AMH and FSH on predicting live birth rates for patients with four combination of age and BMI. **Figure S5.** Estimated generalized additive mixed models (GAMMs) using only first cycle of each patient. A) AMH, and B) FSH. **Figure S6.** Joint effect model of AMH and FSH on predicting live birth rate using only first cycle of each patient. A) 30 year old, B) 35 year old, C) 37 year old, and D) 40 year old. (DOCX 999 kb)

## Abbreviations

AMH: anti-müllerian hormone
FSH: follicle stimulating hormone
GAMM: Generalized additive mixed models
IVF: in vitro fertilization
