# Supplementary material for: Discordant anti-müllerian hormone (AMH) and follicle stimulating hormone (FSH) among women undergoing in vitro fertilization (IVF): which one is the better predictor for live birth?
Source: J Ovarian Res. 2018 Jul 16;11:60. doi: 10.1186/s13048-018-0430-z (PMC6048693; doi:10.1186/s13048-018-0430-z)
Supplement: Supplementary file 1 — Figure S1. Estimated generalized additive mixed models (GAMMs) on different ages without adjusting for centers. A) AMH, and B) FSH. Figure S2. Joint model of AMH and FSH on predicting live birth rates without adjusting for centers. A) 30 year old, B) 35 year old, C) 37 year old, and D) 40 year old. Figure S3. Estimated generalized additive mixed models (GAMMs) on age and BMI. A) AMH, and B) FSH. Figure S4. Joint effect of AMH and FSH on predicting live birth rates for patients with four combination of age and BMI. Figure S5. Estimated generalized additive mixed models (GAMMs) using only first cycle of each patient. A) AMH, and B) FSH. Figure S6. Joint effect model of AMH and FSH on predicting live birth rate using only first cycle of each patient. A) 30 year old, B) 35 year old, C) 37 year old, and D) 40 year old. (DOCX 999 kb) [file 13048_2018_430_MOESM1_ESM.docx]

**Supplementary Files**

**Appendix I: Estimated generalized additive mixed models (GAMMs) on age, AMH, and FSH without adjusting for centers. Figure S1 A and B are two separate marginal models for AMH and FSH, each adjusting for age. Figure S2 A-D shows the joint model of AMH and FSH on predicting live birth rate for patients with age 30, 35, 37, 40.**


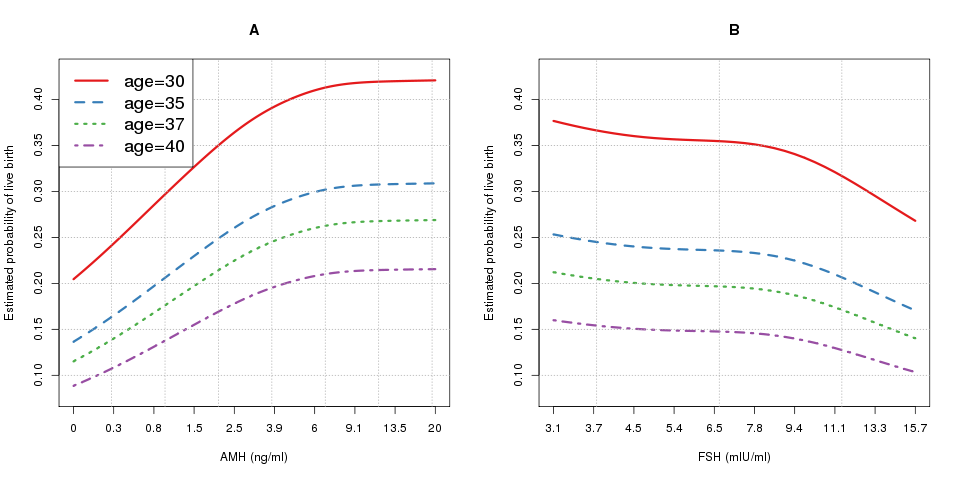


Figure S1


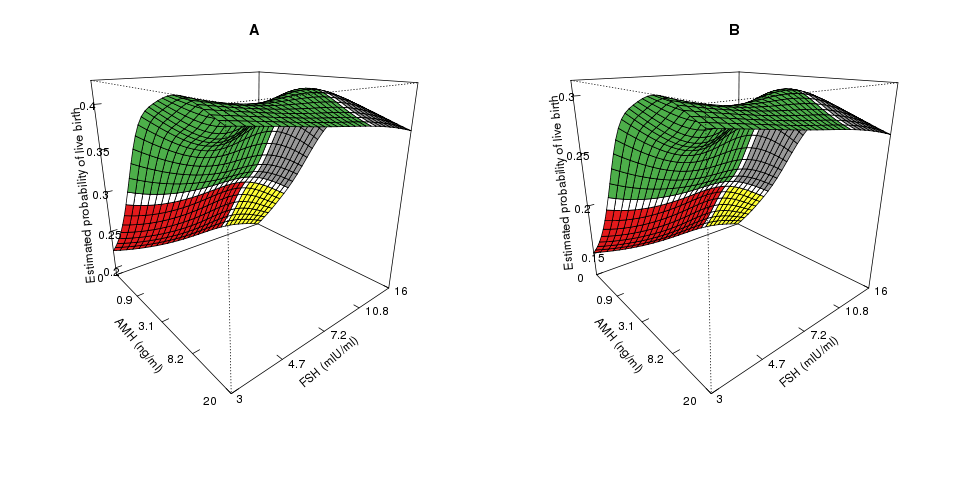


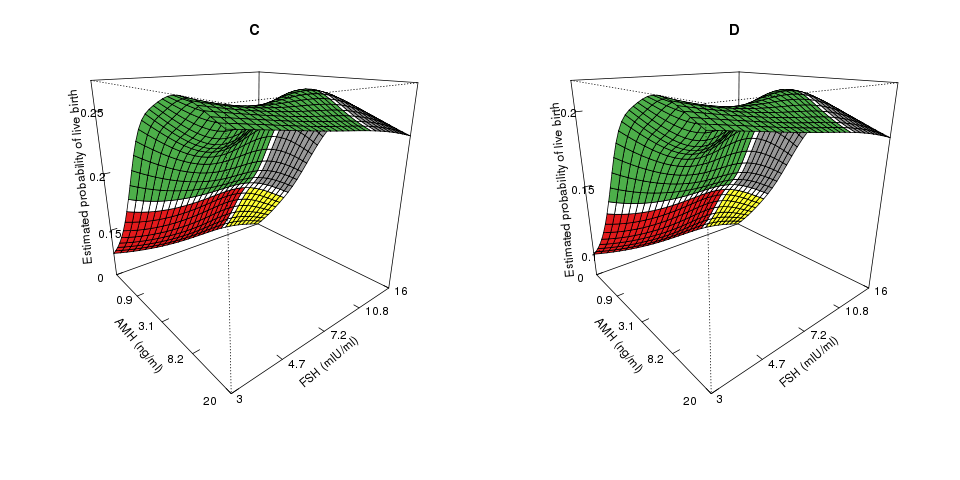


Figure S2

**Appendix II: Estimated GAMMs on age, BMI, AMH and FSH adjusting for centers using the same patient population in the manuscript. Figure S3 A and B are two separate marginal models for AMH and FSH, each adjusting for age and BMI. Figure S4 shows the joint effect model of AMH and FSH on predicting live birth rate for patients with four combinations of age and BMI as marked in the figure legend.**


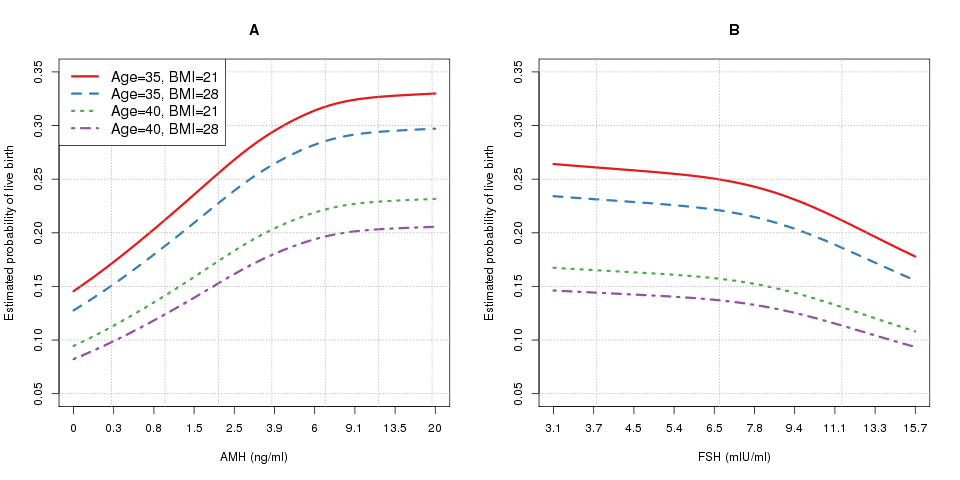


Figure S3


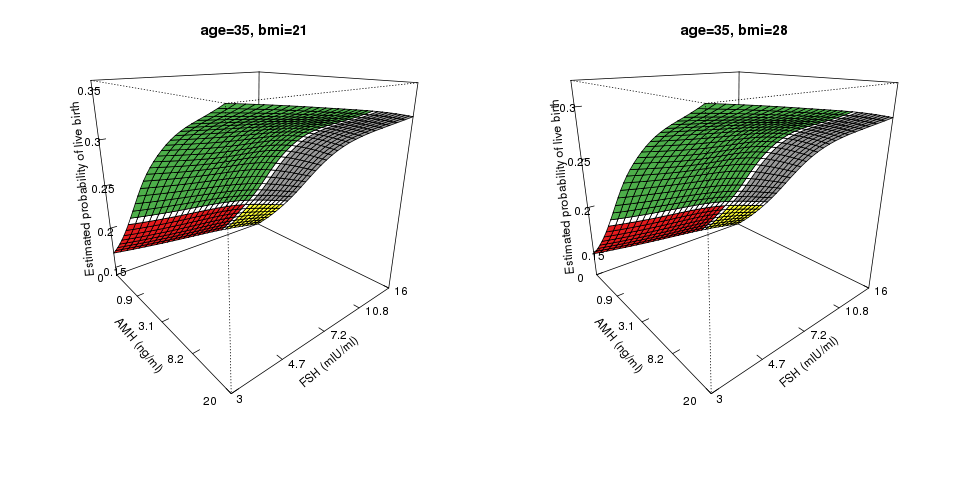


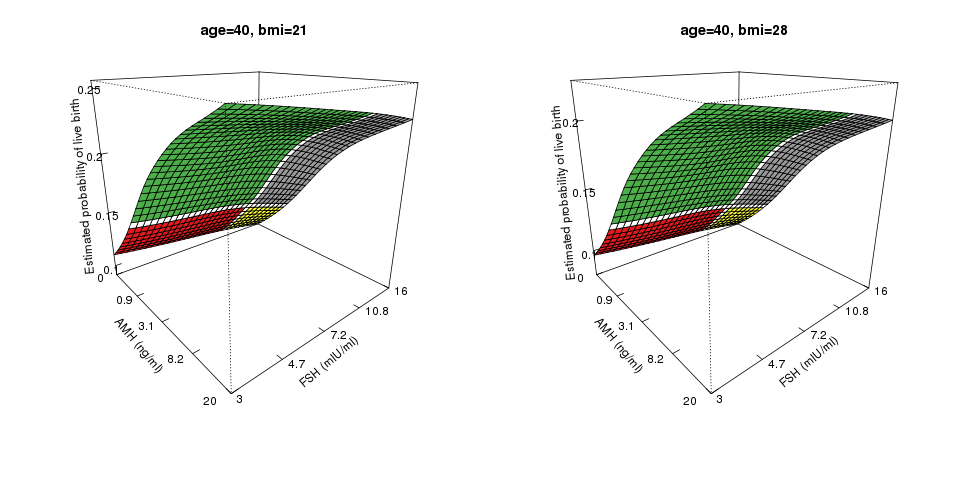
Figure S4

**Appendix III: Estimated GAMMs on age, AMH, and FSH using only the first cycle of each patient, while adjusting for center’s random effect. Figure S5 A and B are two separate marginal models for AMH and FSH, each adjusting for age and center. Figure S6 A-D shows the joint effect model of AMH and FSH on predicting live birth rate for patients with age 30, 35, 37, 40.**


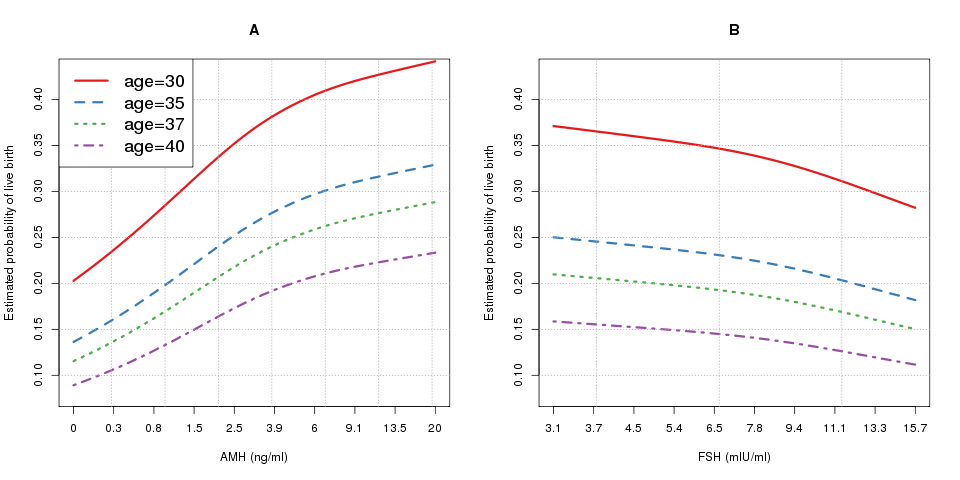


Figure S5


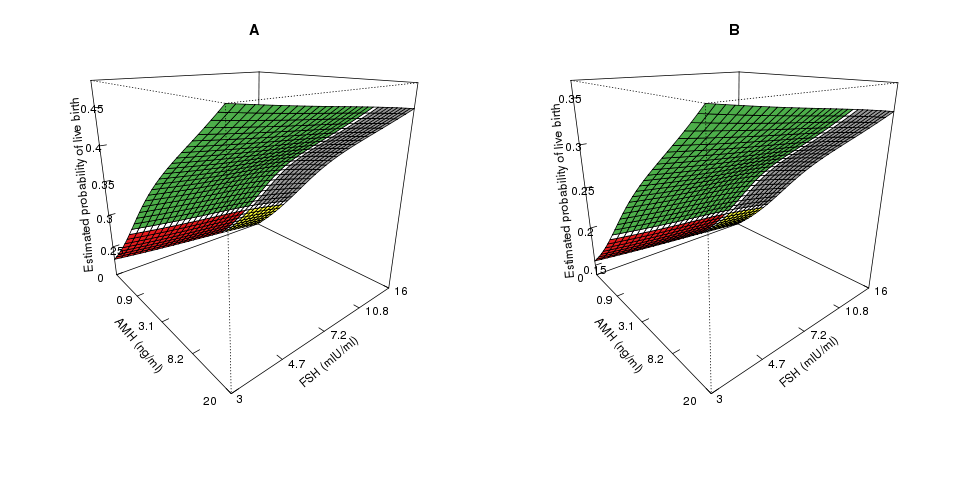


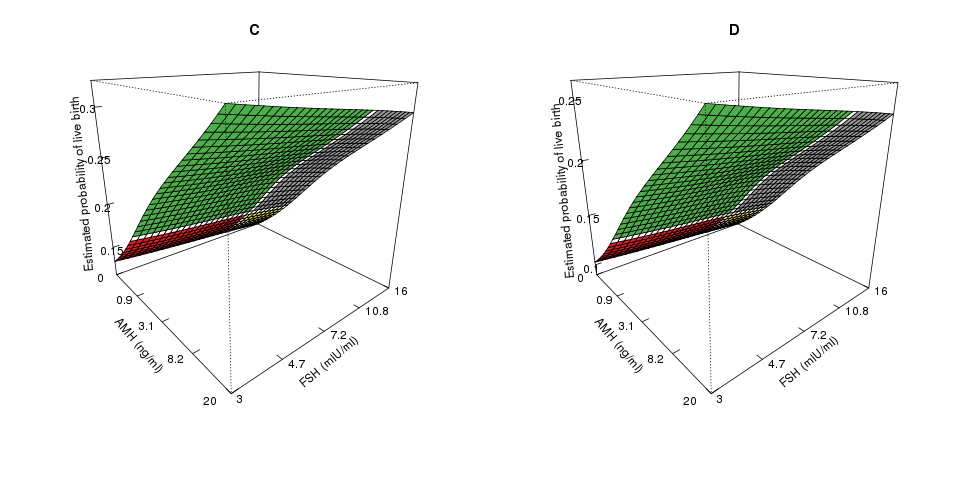


Figure S6

**Appendix IV: The proportions of PCOS, male factor, and protocol types among all four groups**

|  | | **Concordant** | | **Discordant** | |  |
| --- | --- | --- | --- | --- | --- | --- |
|  | **All** | **Group I: Good Prognosis**  **(AMH ≥ 1 & FSH < 10)** | **Group II: Poor Prognosis**  **(AMH < 1 & FSH ≥ 10)** | **Group III: Reassuring FSH**  **(AMH < 1 & FSH < 10)** | **Group IV: Reassuring AMH**  **(AMH ≥ 1 & FSH ≥ 10)** |  |
|  |  |  |  |  |  | **P-values** |
| PCOS* | 6.4% | 9.5% | 1.2% | 2.4% | 3.5% | <0.001 |
| Male Factor* | 15.3% | 16.4% | 12.1% | 14.5% | 15.0% | <0.001 |
| **Protocol** |  |  |  |  |  |  |
| Agonist_long | 22.7% | 29.9% | 6.9% | 14.2% | 18.6% | <0.001 |
| Antagonist | 51.7% | 49.8% | 56.3% | 52.6% | 57.3% |  |
| Microdose | 16.6% | 11.9% | 25.6% | 22.8% | 18.8% |  |
| Natural/Mild | 0.8% | 0.4% | 2.5% | 0.9% | 0.8% |  |

***** There were 48.0% without diagnosis; the proportion was the documented diagnosis among all subjects

Table S1
